# Psychotic and autistic traits among magicians and their relationship with creative beliefs

**DOI:** 10.1192/bjo.2023.609

**Published:** 2023-11-15

**Authors:** Gil Greengross, Paul J. Silvia, Sara J. Crasson

**Affiliations:** Department of Psychology, Aberystwyth University, Wales; Department of Psychology, University of North Carolina at Greensboro, USA; Flavors of Magic, New York, USA

**Keywords:** Magic, creativity, schizophrenia, autism, psychoticism

## Abstract

**Background:**

There is a common perception that creativity is associated with psychopathology. Previous studies have shown that members of creative groups such as comedians, artists and scientists scores higher than the norm on psychotic traits, and scientists in STEM (science, technology, engineering and mathematics) fields score highly on autistic traits.

**Aims:**

To test whether magicians, a creative group that has not been studied before, also score highly on psychopathological traits and autism, and to test the associations of creative self-efficacy and creative identity with schizotypal and autistic traits among magicians.

**Method:**

A sample of 195 magicians and 233 people from the general population completed measures of schizotypal traits (Oxford–Liverpool Inventory of Feelings and Experiences) and autism (Abridged Version of the Autism-Spectrum Quotient), as well as the Short Scale of Creative Self. Magicians were also compared with other creative groups with respect to schizotypal traits, based on previously published data.

**Results:**

Magicians scored lower than the general population sample on three of the four schizophrenia measures (cognitive disorganisation, introvertive anhedonia and impulsive nonconformity) but did not differ with respect to unusual experiences or autism scores. Magicians scored higher on creative self-efficacy and creative personal identity than the general sample. Magicians’ scores on schizotypal traits were largely lower than those of other creative groups. Originality of magic was positively correlated with unusual experiences (*r* = 0.208), creative self-efficacy (*r* = 0.251) and creative identity (*r* = 0.362).

**Conclusions:**

This is the first study to show a creative group with lower scores than norms on psychotic traits. The results highlight the unique characteristics of magicians and the possible myriad associations between creativity and mental disorders among creative groups.

Creativity is often viewed as a highly desired trait, with many people wishing to be more creative.^1^ It is commonly thought that there is a connection between creativity and mental illness, and that having a mental disorder could boost creativity.^2^ Growing evidence shows that creativity is indeed associated with psychopathology, although the nature of this association is not fully understood.^3,4^ Much of the research has focused on the connection between schizophrenia and creativity, with studies yielding mixed results.^5–7^ Some of the discrepancies in results may be explained by researchers using different definitions and measures of schizophrenia. For example, studies focusing on subclinical cases of schizophrenia and those measuring proclivity to schizotypal behaviours in the general population without a clinical diagnosis of the disorder tend to find positive correlations between creativity and schizophrenia. On the other hand, studies looking at people clinically diagnosed with schizophrenia yield mostly negative correlations, suggesting that the true relationship between creativity and schizophrenia is nonlinear and closer to an inverted U.^7^ Schizotypy is the most common construct used to assess individual differences on the schizophrenia spectrum.^4,8^ The measure is not intended to diagnose schizophrenia, and individuals scoring highly on schizotypal traits are not necessarily schizophrenic but rather display behaviours and share experiences that might be indicative of the disorder. Various studies have found higher levels of schizotypal traits (particularly positive schizotypy – excessive perceptual experiences and thoughts, which have been hypothesised to help generate creative and original ideas)^9^ among members of creative vocational groups such as artists, comedians and scientists.^10–15^ The current research focuses on one distinct creative group with unique characteristics that has not been studied before – magicians.

## Magic

Magic performances have been popular throughout history and are prevalent across cultures.^16–18^ As both creators and performers of magic, magicians encompass various creative domains.^19^ From close-up magic that necessitates only simple objects, such as coins and cards, to large illusions that require equipment, large spaces and the support of numerous assistants, magic tricks are highly variable and are a manifestation of different skills. Creative domains differ in the skills they require and their barriers for entry, so different creative professions attract and select for different clusters of traits and abilities.^20^ One thing that distinguishes magicians from most other performing artists is the precision required in their performances. Thus, compared with other performers, it is more difficult to overcome errors. A comedian that botches a joke has plenty of opportunities to tell other jokes and make the audience laugh. A musician that played the wrong note can move on and still play well the rest of the concert. By contrast, although magic performances are highly variable and can include unseen errors, magic tricks are largely ‘all or nothing’ acts that culminate in an ‘aha’ moment of surprise and awe. Failed magic tricks leave a greater impact than unfunny jokes and are harder to compensate for, as they are few and far between. Thus, in addition to requiring highly technical skills, regardless of the type of magic performed, the high stakes of magic performances make magicians a unique creative group to study among all artistic professions. Studying magicians could therefore further shed light on the link between creativity and psychotic traits. There is also a commonly held belief among magicians that male magicians come to magic to make up for a social deficit. This is another basis for the hypothesis that magicians will score higher on autistic/psychotic traits.

## Aims of the study

We used the Oxford–Liverpool Inventory of Feelings and Experiences (O-LIFE)^8,21^ to compare schizotypy traits among magicians with a sample matched for age and sex from the general population. We also conducted a secondary analysis to compare the magicians’ scores on O-LIFE with those of other creative groups whose scores had been previously published. These groups included comedians,^13^ actors,^13^ musicians,^11^ poets,^12^ and artists (mostly visual).^22^^a^

In addition, we tested whether magicians differed from the general population on autism spectrum traits, another mental disorder that is often associated with creativity.^23^

Based on previous studies of other creative groups, we hypothesised that magicians would have higher scores on psychotic and autistic traits, compared with a sample from the general population. Last, we looked at magicians’ beliefs in their own creative ability and how big a part creativity plays in their identity, including how these differ compared with those of the general population and how they relate to psychotic traits and autism.^24^

## Method

### Design and participants

The design of the study was correlational, comparing a sample of magicians and a sample taken from the general population on measures of psychoticism, autism and creative beliefs. Magicians were invited to take part in the study through newsletters and publications of the Society of American Magicians, International Brotherhood of Magicians, Magic Circle or Academy of Magical Arts, as well as through magic groups on social media. All magicians’ contacts were made by the third author, who served at the time as the Resourceress of the Society of American Magicians. We aimed to sample as many magicians as possible, and there was no limit on the number of magicians recruited for the study. Recruitment took place between December 2020 and January 2021. As the number of female magicians was small, another letter targeting female magicians was sent in August 2021. A general sample was obtained from the research platform Prolific.co, matching the magicians’ sample for age and including a similar proportion of men and women. All participants answered basic demographic questions about their age, sexual identity, education, marital status and ethnicity. All participants were 18 years old or older and completed the study online. We assert that all procedures contributing to this work comply with the ethical standards of the relevant national and institutional committees on human experimentation and with the Helsinki Declaration of 1975, as revised in 2008. All procedures involving human subjects were approved by the Department of Psychology, Aberystwyth University, with approval number 16 579. All participants consented to participate in the study by ticking a box in the online study. Responses were fully anonymised by the authors.

### Measures

- Short version of the O-LIFE inventory.^8^ The questionnaire includes four subscales that measure schizotypy in non-clinical populations. The subscales are as follows.
  - Unusual experiences (12 items); includes positive schizotypy symptoms such as experiences of perceptual aberrations, magical thinking and hallucinations.
  - Cognitive disorganisation (11 items); involves the inability to focus or concentrate, inability to control thoughts, and social anxiety.
  - Introvertive anhedonia (ten items); contains negative schizotypy symptoms such as lesser ability to enjoy physical and intimate pleasure, and avoidance of intimacy.
  - Impulsive nonconformity (ten items); includes impulsive, antisocial, and spiteful thoughts and behaviours, often indicating low self-control.
- Abridged Version of the Autism-Spectrum Quotient^25^ (ten items). This scale measures autistic traits in non-clinical populations without any learning disability.
- Short Scale of Creative Self.^24^ The instrument consists of the following two subscales.
  - Creative self-efficacy (six items); measures the belief that an individual has in their ability to be creative.
  - Creative personal identity (five items); measures how much being creative is part of one's own identity.
- Originality of magic. Magicians estimated how original their magic was, from 0 (‘everything I perform is done with a purchased trick, or as described in a book or magazine, with the script provided by the inventor’) to 100 (‘everything I perform is completely my own creation and script, and I build all my own props’).

## Results

**Fig. 1:**
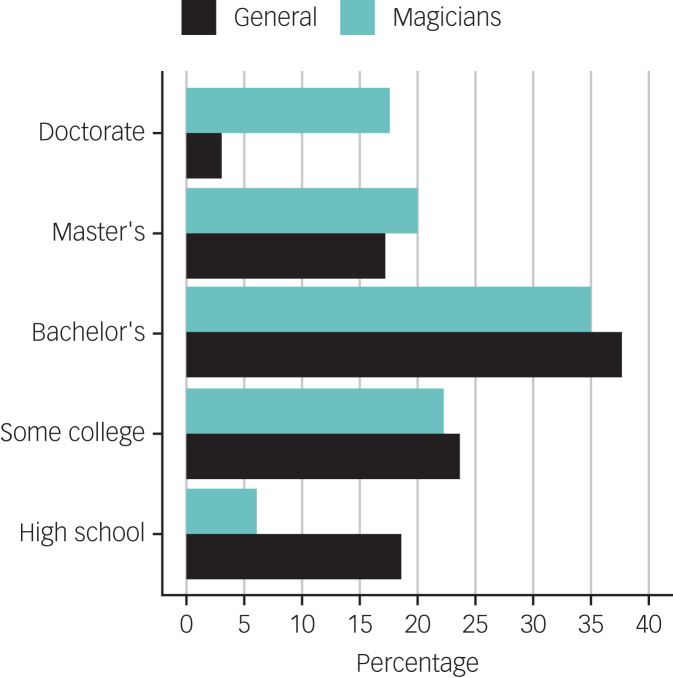
Highest levels of education for magicians and general population samples.

The sample of magicians included 195 individuals (164 men, 30 women, one non-response) with a mean age of 56.59 years (s.d. = 16.59; range 18–90). The general population sample included 223 individuals (183 men, 37 women, one other, two non-responses), with a mean age of 55.30 years (s.d. = 15.31; range 20–93). There were no age differences between the two groups (*t* = 0.82, *P* = 0.413). The majority of both samples identified as White (89.6% of magicians and 96.8% of those answering the question). The highest levels of education for both magicians and the general samples are shown in Fig. 1.

Magicians’ experience in doing magic ranged from 1–79 years, with an average of 34.69 years (s.d. = 20.59). The type of magic the magicians did varied, with 77% doing close-up magic, 69% parlour/platform magic, 59% card magic, 42% mentalism, 32% stage magic, 3% large illusions and 3% cardistry (answers are not mutually exclusive). The main places where magicians performed (not exclusively) were cocktail/birthday parties (65%), theatres (37%), corporate events (31%), schools/colleges (26%), street magic/fairs (25%) and comedy clubs/shows (25%). The average originality of magic score was 45.14 (s.d. = 26.21; range 0–100).

Mean scores for the two samples, independent *t*-tests comparing the differences and Cohen's *d* effect sizes for the O-LIFE, autism and creative measures are presented in Table 1. Magicians scored significantly lower on three of the four psychotic subscales (cognitive disorganisation, introvertive anhedonia and impulsive nonconformity) and higher on the two creative measures compared with the general sample. There were no significant differences between the groups for unusual experiences and autism.

**Table 1:** Means, standard deviations, *t*-tests and effect sizes^a^ comparing magicians (*n* = 195) and the general population (*n* = 223) on the Oxford–Liverpool Inventory of Feelings and Experiences, autism, creative self-efficacy and creative personal identity scales

	Group	*N*	Mean (s.d.)	*t* (d.f.)	*P*	Cohen's *d* (95% CI)
Unusual experiences	Magicians	195	3.16 (2.71)	−1.77 (357)	0.078	−0.19 (−0.40, −0.02)
	General	164	3.64 (2.34)			
Cognitive disorganisation	Magicians	195	3.02 (2.44)	−7.41 (383)	<0.001	−0.76 (−0.96, −0.55)
	General	190	5.09 (3.02)			
Introvertive anhedonia	Magicians	195	2.50 (2.00)	−7.01 (391)	<0.001	−0.71 (−0.91, −0.50)
	General	198	3.96 (2.14)			
Impulsive nonconformity	Magicians	195	2.04 (1.67)	−3.83 (376)	<0.001	−0.39 (−0.60, −0.19)
	General	183	2.69 (1.62)			
Autism	Magicians	195	3.23 (1.91)	−0.39 (406)	0.695	−0.04 (−0.23, 0.16)
	General	213	3.31 (1.92)			
Creative self-efficacy	Magicians	187	4.13 (0.59)	7.34 (408)	<0.001	0.73 (0.53, 0.93)
	General	223	3.65 (0.71)			
Creative personal identity	Magicians	187	4.24 (0.77)	10.22 (408)	<0.001	1.01 (0.81, 1.22)
	General	223	3.29 (1.06)			

a. Positive effect size denotes that magicians scored higher than the general population on the scale.

To test whether sex was a moderating factor, we ran multiple analyses of covariance for each of the scales, with group and sex as fixed factors. None of the analyses revealed a significant interaction effect between group and sex; thus, sex did not seem to be a moderating factor and was excluded from further analyses.

Table 2 shows the intercorrelations among O-LIFE, autism and creative scale scores for both magicians and the general population. The correlations of magicians’ scores on these scales with originality of magic scores are also shown. Originality of magic was moderately correlated with unusual experiences (*r* = 0.208), creative self-efficacy (*r* = 0.251) and creative personal identity (*r* = 0.362). In addition, for magicians only, unusual experiences scores were moderately correlated with scores on both creative subscales (*r* = 0.386 for creative self-efficacy and *r* = 0.334 for creative personal identity).

**Table 2:** Intercorrelations for magicians (below the bold diagonal) and for the general population (above the bold diagonal) among originality of magic, O-LIFE, autism, creative self-efficacy and creative personal identity scales; Cronbach's α reliability coefficients for all participants on all scales are on the bold diagonal

Variable	Original^a^	UnEx	CogDis	IntAn	ImpNon	Autism	CSE	CPI
Original	**_**	**_**	**_**	**_**	**_**	**_**	**_**	**_**
UnEx	0.208**	**0**.**78**	0.198*	−0.062	0.260**	0.004	0.151	0.112
CogDis	−0.027	0.179*	**0**.**81**	0.304***	0.452***	0.275***	−0.230***	−0.126
IntAn	−0.033	0.027	0.320***	**0**.**66**	0.034	0.178*	−0.217**	−0.122
ImpNon	−0.014	0.267***	0.384***	0.078	**0**.**53**	0.090	0.102	0.145
Autism	−0.055	−0.039	0.313***	0.097	0.104	**0**.**52**	−0.210**	−0.085
CSE	0.251***	0.386***	−0.050	−0.109	0.036	−0.197**	**0**.**85**	0.768***
CPI	0.362***	0.334***	0.037	−0.188***	0.041	−0.110	0.691***	**0**.**94**

UnEx, unusual experiences; CogDis, cognitive disorganisation; IntAn, introvertive anhedonia; ImpNon, impulsive nonconformity; CSE, creative self-efficacy; CPI, creative personal identity.

a. Original scores are for magicians only.

*P < 0.05, **P < 0.01, ***P < 0.001.

**Fig. 2:**
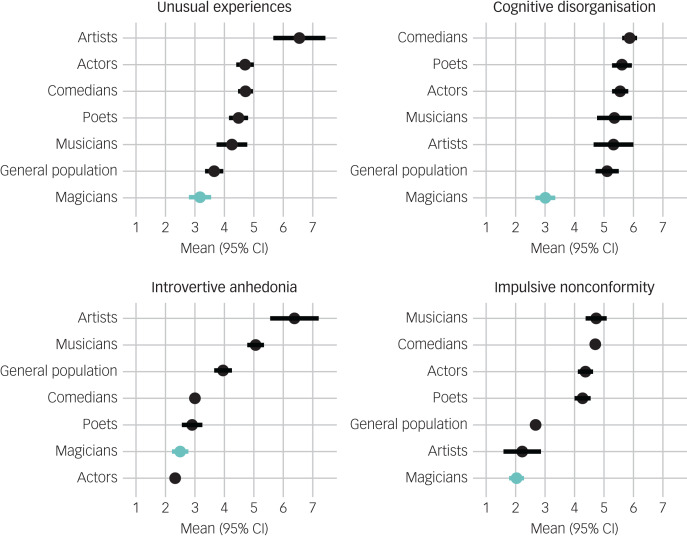
Comparison of the four Oxford–Liverpool Inventory of Feelings and Experiences scales (unusual experiences, cognitive disorganisation, introvertive anhedonia and impulsive nonconformity) for various creative groups and the general sample.

Figure 2 shows the O-LIFE scores of magicians compared with those of comedians, actors, musicians, poets, artists and the general sample. One-way analysis of variance for each psychotic measure showed significant differences (*P* < 0.001 for all four). We followed up with Tukey HSD *post hoc* tests comparing the magicians’ scores on each subscale with those of all other groups. The results showed that magicians scored significantly lower than all other creative groups on unusual experiences (*P* < 0.05 compared with musicians; *P* < 0.001 compared with all other groups) and on cognitive disorganisation (*P* < 0.001 for all groups). For introvertive anhedonia, magicians scored significantly lower only compared with artists and musicians (*P* < 0.001). Last, magicians scored significantly lower than all groups except artists on impulsive nonconformity (*P* < 0.001). Notably, magicians did not score higher than any other creative group on any of the O-LIFE sub-scales. Cohen's *d* effect sizes for all pairwise comparisons on all O-LIFE measures are shown in Table 3.

**Table 3:** Cohen's *d* effect sizes^a^ for all pairwise comparisons between magicians and other creative groups on the four O-LIFE sub-scales

	Comedians	Actors	Artists	Poets	Musicians
Unusual experiences	−0.55	−0.55	−1.22	−0.48	−0.40
Cognitive disorganisation	−1.07	−0.97	−0.98	−0.97	−0.86
Introvertive anhedonia	−0.25	0.09	−1.63	−0.16	−1.44
Impulsive nonconformity	−1.35	−1.34	−0.11	−1.00	−1.56

a. Positive effect size denotes that magicians scored higher than the general population on the scale.

## Discussion

Our study demonstrates that not all creative people are created equal. Magicians scored significantly lower than the general population and other vocational creative groups on most psychopathological traits, particularly those related to schizotypy.^11–14,22^ The notion that creative people have high levels of psychotic traits, or that a proclivity to psychoticism is associated with higher creative output, is prevalent and generally supported by research. For example, people diagnosed with schizophrenia have been found to be more likely to work in creative jobs.^5^ However, our study demonstrates that the relationship between creativity and psychoticism is more complex and likely to be dependent on the nature of the creative work and the specific skills and characteristics needed to succeed in it.

Magicians scored lower than the general sample and other creative groups on cognitive disorganisation. People scoring high on cognitive disorganisation find it hard to concentrate and are more likely to suffer from social anxiety. Magicians’ work requires focus, attention to detail and composure, and lacking such traits would be counterproductive and detrimental to their performance.

Magicians also scored low on impulsive nonconformity, a trait that is associated with antisocial behaviour and lower self-control. These traits are valuable for many creative groups such as writers, poets and comedians, whose creative acts are often edgy and challenge conventional wisdom. Magicians can be equally innovative and push the limits of what is thought to be possible in magic (e.g. David Copperfield's famous flying illusion); however, many magicians perform familiar tricks or some variations of them without feeling the need to innovate. The magician's oath not to reveal the secrets behind the tricks preserves the mystery and surprise, and creative performers can use those ideas and techniques over and over again, to the delight of the audience. Magicians’ on-stage personalities are also generally affable, as they often need the cooperation of the audience or the help of assistants in performing their magic acts. Having high levels of self-control and being less impulsive are also valuable in magicians’ performances, as they need to be very precise in their work for the tricks to go well. Impromptu acts in magic are rare. Although a presentation may include some improvisation, the technique is usually well rehearsed. If the technique has to be changed during the show for any reason, it often suggests that things are not proceeding as intended.

Notably, although magicians did not differ from the general population on the unusual experiences subscale, they scored lower on that scale than other creative groups. Higher levels of this type of positive schizotypy – having hallucinations and excessive perceptual experiences, and the belief in magical thinking – has been reported among other artists such as poets,^12^ musicians^11^ and visual artists.^15,22^ This type of schizotypy may be less relevant to magicians’ work, as it is likely to interfere with their performance, which requires discipline and focus.

However, magicians who did score highly on unusual experiences reported being more original in their magic performances and had stronger convictions about their creative abilities, and creativity was a bigger part of their identity. This somewhat contradictory role of unusual experiences in magicians’ life may be due to the multiple dimensions of magicians’ work. What distinguishes magicians from most other creative people is that they not only create their own magic tricks but also perform them, whereas most creative groups are either creators or performers. For example, poets, writers, composers and choreographers create something that will be consumed or performed by others. By contrast, actors, musicians and dancers perform and interpret the creations of others.^26^ Magicians, like comedians and singer–songwriters, are among the rare groups that do both. Although not all magicians invent the tricks they perform, magicians still need to adapt the trick to their own style, practise it rigorously and perform it with meticulous precision with little room for error – all while simultaneously entertaining the audience. Magicians are also unique in that there are many types of magic illusion, each requiring a different set of skills and dedicated training. Card tricks, mentalism and large illusions are quite distinct forms of magic, making the creative output of magicians among the most diverse and heterogeneous of all creative acts.

Magicians did not differ from the general population on autistic traits. Although magicians did not score significantly lower on this scale, as they did with most of the schizotypal traits, the absence of high autism scores suggests a lack of disposition for both mental and neurodevelopmental disorders among magicians.

The relatively low scores of magicians on schizotypal and autistic traits may be beneficial for their work, as a proclivity to psychotic and autistic traits could be counterproductive for magicians and magic shows. Although magicians often work in isolation when creating and practising their magic, they also regularly collaborate and socialise with others on and off the stage. Magicians work with other magicians on developing their tricks, coach each other and brainstorm together, and they may use assistants to perform on stage. There is also the business side of magic, where magicians have to find venues to perform, negotiate their fees with club managers, often travel large distances – all requiring various social skills, careful attention and focus. It is much harder to negotiate all these intricacies for magicians who score highly on psychotic and autistic traits. Thus, it is possible that magicians self-select into their profession, with aspiring magicians with higher levels of psychotic and autistic traits not being very successful and dropping out, and magicians with traits that offer the best chance of success thriving. In addition, there is no formal magic education, and aspiring magicians, at least traditionally, had to find a teacher and maintain a relationship with them to get a magic education. People with high levels of psychotic and autistic traits may face more difficulties in finding and maintaining such relationships.

Magicians’ schizotypy profile seemed to be most similar to those of mathematicians and scientists. In one study, mathematicians scored significantly lower than non-mathematicians (sample including the general population, psychiatric patients, poets and visual artists) on unusual experiences, cognitive disorganisation and impulsive nonconformity but did not differ on introvertive anhedonia.^14^ Another study found that biological and physical scientists scored lower than visual artists and musicians on unusual experiences and lower than musicians on cognitive disorganisation, whereas they did not differ from either group on impulsive nonconformity or introvertive anhedonia.^10^ The results of both of these studies are consistent with magicians’ low scores on schizotypy traits in our study. The orderliness and persistence associated with the work of scientists may be compared to the work of magicians, who need to practise scrupulously before they go on stage, where they need to be very accurate in their performance. Moreover, just like scientists, magicians endeavour to achieve a specific goal built on many small steps that often can be reached in multiple ways, with varying levels of creativity. Such concentrated effort and dedication are often also associated with higher levels of autistic traits, something that is found among mathematicians and scientists^27^ but was not found for the magicians in our study.

Magicians had stronger views of themselves as creative people and saw creativity as a more important part of their identity, compared with the general sample. It is likely that people who are not part of any creative group do not think much about creativity in their daily lives; thus creativity, is not a big part of their identity. Interestingly, whereas magicians showed significant correlations between the creativity measures and unusual experiences, no such correlations existed among the general population. Perhaps, people not belonging to any particular creative group do not think much about their creative abilities and thus do not make such a connection. By contrast, magicians, as creators and performers of magic, are more conscious of their creative abilities and can see how unusual experiences relate to them. Being more aware of their own creativity might make magicians more creative. In support of such a connection, we found a small correlation between originality of magic and unusual experiences. We also found small to medium correlations between originality of magic and the two creative measures, meaning that more creative magicians also had stronger beliefs about their creative capabilities and saw creativity as a big part of who they are.

In sum, our study found that the widely reported connection between creativity and mental illness may be more complex than previously thought and may depend on the type of creative work that is pursued. Magicians in our study scored lower on most schizotypy traits compared with a general sample, in contrast to what has been found in studies on other artistic groups. Magicians have distinct characteristics and experiences compared with other people, and with other artistic groups; they are more similar to scientists, which makes them a unique creative group worth further study.^19,28,29^ Our findings are also consistent with anecdotal evidence of the lack of prevalence of mental disorders among notable magicians, whereas there are plenty of examples among other creative occupations. Some of the famous cases include comedians (Robin Williams and Sarah Silverman), poets (Sylvia Plath), writers (Virginia Woolf), painters (Van Gogh and Georgia O'Keeffe), singers (Brian Wilson and Billie Eilish) and scientists (Kurt Gödel and John Nash). There is a common perception among laypeople and clinicians alike that many creative people have mental illnesses, and that such illnesses make them more creative.^2^ Our research shows that members of at least one creative group, magicians, do not exhibit higher levels of mental disorders. This finding may be of interest to clinicians in developing new interventions, as it demonstrates that the association between creativity and psychopathology is more complex than previously thought, and that different types of creative work could be associated with either high or low levels of psychoticism or autistic traits.

### Limitations and future research

Our study included a relatively older sample of magicians that may not represent all practising magicians. Most magicians were members of the Society of American Magicians or other magic clubs, and perhaps younger magicians are less likely to be part of magic organisations. Although magicians were matched for age with the general sample, the average age of magicians was higher than those of other creative groups for whom data on psychotic traits exist.^10–12,14,15,22^ Our study was also based on self-reports and so we cannot draw conclusions on causal relationships between creativity and mental illness. Although the scales used in this study are commonly used by researchers and considered reliable and valid, originality of magic was assessed based on self-report and was not evaluated independently.

Future research should aim to compare various creative groups and individuals in STEM (science, technology, engineering and mathematics) fields with a sample from the general population, in one study using the same methods and tools. That way, we will be able to get a more reliable and complete picture of the differences between creative subtypes. The magicians sample included a relatively low number of women, despite specific efforts to recruit as many women magicians as possible. Although sex was not a moderating factor in our analysis, this skewed sex ratio raises interesting questions about the role of women in magic. There are several creative groups where women are a minority, perhaps most notably stand-up comedians.^30,31^ Future studies could look at the causes of such a sex disparity, and whether men and women magicians differ from each other and how they perform on stage.

## Data Availability

The study data are available upon reasonable request from the corresponding author.

## Footnotes

a These groups used the same version as that used in the current study. Additional creative groups with O-LIFE scores from a different version were excluded.
